# Comparative Biochemistry of Wild and In Vitro-Propagated *Melilotus officinalis*: Phytochemical Composition, Antioxidant, and Antimicrobial Properties

**DOI:** 10.3390/life16091488

**Published:** 2026-09-05

**Authors:** Gulmira Zhakupova, Ângela Liberal, Assem Sagandyk, Tayse F. F. da Silveira, Tania Pires, Aigerym Akhmetzhanova, Aknur Muldasheva, Anastassiya Tyurina, Lillian Barros

**Affiliations:** 1 Saken Seifullin Kazakh Agrotechnical University, Astana 010000, Kazakhstan; 2 CIMO, LA SusTEC, University Polytechnic de Bragança, Campus de Santa Apolónia, 5300-253 Bragança, Portugal; 3 Research Center for Active Living and Wellbeing (LiveWell), University Polytechnic de Bragança, Campus de Santa Apolónia, 5300-253 Bragança, Portugal

**Keywords:** medicinal plants, secondary metabolites, phenolic profile, organic acids, antioxidant assays, antimicrobial assays, in vitro culture, Kazakhstan flora

## Abstract

*Melilotus officinalis* (L.) Lam. (yellow sweet clover) is a medicinally valuable Fabaceae species with recognized antioxidant and antimicrobial properties, yet the impact of cultivation system on its secondary metabolism remains poorly characterized for Central Asian populations. This study compared wild and in vitro-grown *Melilotus officinalis* from northern Kazakhstan (Astana city), examining phenolic composition, organic acids, antioxidant capacity, and antimicrobial activity as functions of cultivation system. Hydroethanolic extracts were analyzed by HPLC-DAD-ESI-Orbitrap MS/MS and UFLC-PDA, while antioxidant activity was assessed by TBARS and ABTS assays and antimicrobial activity by broth microdilution against eight bacterial and two fungal strains. Thirteen phenolic compounds were tentatively identified, revealing higher total flavonoid content in in vitro plants, dominated by apigenin di-C-pentoside isomers and vicenin-3, whereas wild plants showed greater diversity of phenolic acids and flavonols, including kaempferol and quercetin glycosides. Despite lower total phenolics, wild extracts more effectively inhibited lipid peroxidation, while ABTS radical scavenging was comparable between sources, and antimicrobial effects were strain-specific. Oxalic acid was approximately three-fold higher in in vitro material, a biochemical feature relevant for downstream use. These findings indicate that cultivation system substantially shapes the secondary metabolite profile and bioactivity of this regionally important medicinal species.

## 1. Introduction

Oxidative stress, resulting from an imbalance between the production of reactive oxygen species (ROS) and antioxidant defenses, plays a central role in plant and human physiology, contributing to plant stress responses as well as to the onset of chronic diseases in humans, including cardiovascular disorders and neurodegenerative diseases. Plant-derived antioxidants have consequently attracted growing interest as multifunctional bioactive compounds, with potential applications extending from human health to food and pharmaceutical industries [1,2].

Medicinal and aromatic plants are especially relevant in this context due to their richness in secondary metabolites with documented antioxidant activity. Among these, *Melilotus officinalis* (L.) Lam (yellow sweet clover), a biennial species of the Fabaceae family native to Eurasia and naturalized in parts of Europe, Asia, and the Americas, has attracted increasing attention [3]. In Kazakhstan, *Melilotus officinalis* grows widely in steppe and forest-steppe zones, including the northern regions, yet its phytochemical characterization under local wild and controlled cultivation conditions remains largely unexplored. Traditionally used in herbal medicine for its anti-inflammatory, sedative, and vasoprotective effects, this species has been widely employed in the treatment of venous insufficiency and inflammatory conditions [4]. Recent scientific studies have corroborated these traditional applications, highlighting its antioxidant, antimicrobial, anti-inflammatory, and cardioprotective properties [5,6,7].

The biological activity of *Melilotus officinalis* is mainly attributed to its diverse phytochemical composition, which includes coumarins (e.g., coumarin and melilotoside), flavonoids (such as luteolin, kaempferol, and quercetin derivatives), and phenolic acids (including chlorogenic, protocatechuic, and vanillic acids) [7,8]. These compounds are known to act as effective radical scavengers, metal chelators, and modulators of oxidative pathways. Importantly, such phytochemicals also contribute to the functional properties of foods, supporting the development of nutraceuticals and functional ingredients with added health benefits [2].

The concentration and profile of these bioactive compounds are highly influenced by both intrinsic (genetic) and extrinsic factors, including environmental conditions, soil composition, climate, and agronomic practices [7,9]. Wild plants are often exposed to abiotic and biotic stresses that can enhance the biosynthesis of defensive secondary metabolites, potentially increasing their antioxidant capacity [10]. In contrast, in vitro culture techniques offer a controlled environment that enables the standardization of biomass production, the manipulation of metabolic pathways, and the consistent production of target compounds. Moreover, biotechnological approaches, including tissue culture and elicitation strategies, have been increasingly explored to improve the yield and reproducibility of plant-derived metabolites [11].

From a safety perspective, the application of *Melilotus officinalis* in food systems requires careful monitoring, particularly due to the presence of coumarin, a compound associated with hepatotoxicity at high intake levels. Regulatory authorities have established tolerable daily intake (TDI) limits for coumarin, making it essential to balance functional efficacy with safety [12]. Therefore, understanding how cultivation systems (wild versus in vitro) affect both phytochemical composition and compound stability is critical for ensuring safe and effective use.

In this context, the present study aimed to compare the phytochemical profile, antioxidant activity, and antimicrobial activity of wild-collected and in vitro-grown *Melilotus officinalis* from Kazakhstan. The study further aimed to describe cultivation-associated differences in metabolite composition and to explore the potential valorization of this medicinal species as a source of bioactive ingredients.

## 2. Materials and Methods

### 2.1. Sample Preparation

**Figure 1:**
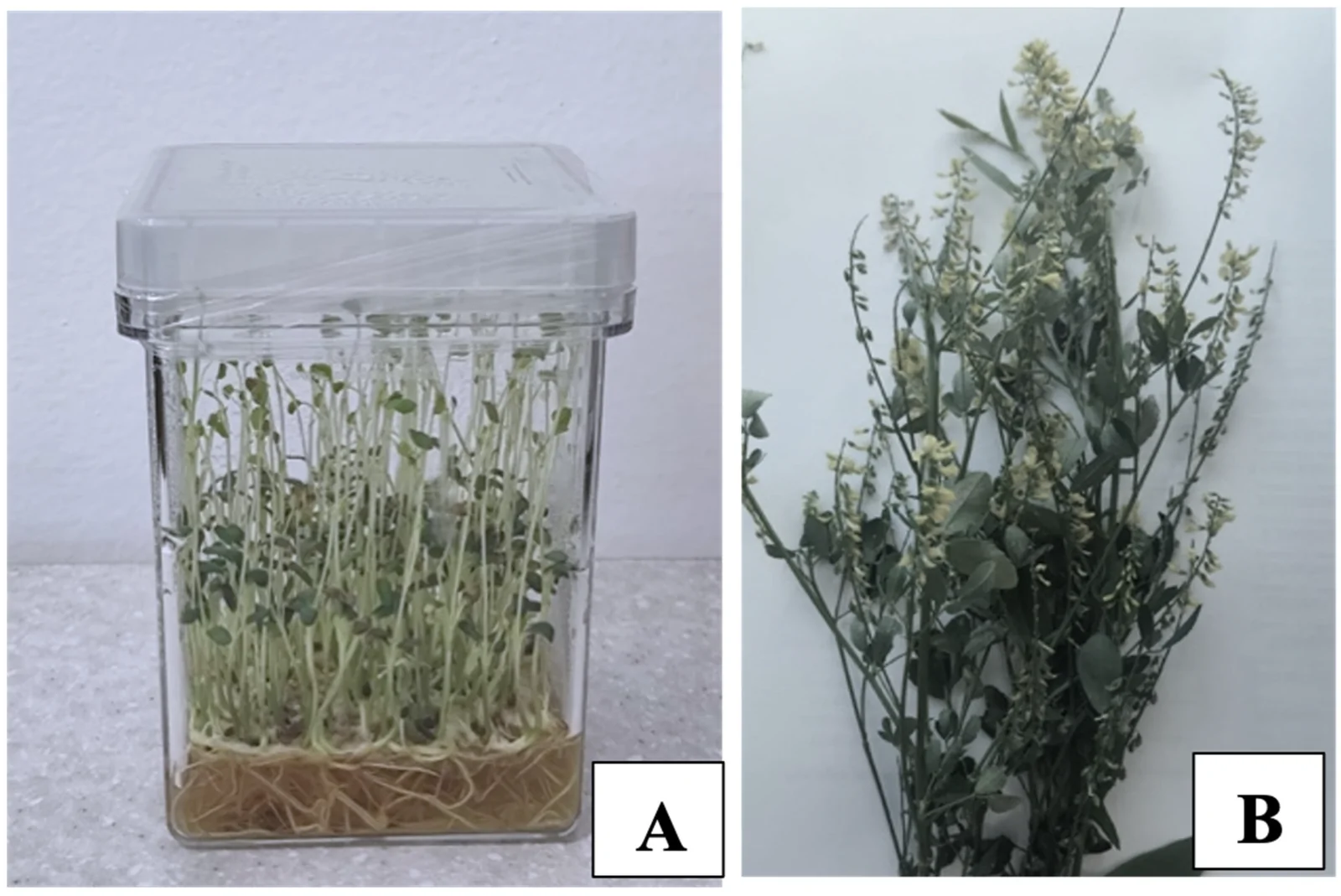
Samples of sweet clover (*Melilotus officinalis* (L.) Lam.): (**A**) in vitro samples; (**B**) wild samples.

Seeds of sweet clover (*Melilotus officinalis* (L.) Lam.; Figure 1) were obtained from the Institute of Engineering and Food Technology, Saken Seifullin Kazakh Agrotechnical University (Astana, Kazakhstan). For the in vitro experiment, plant explants were cultured on Murashige and Skoog (MS) basal medium (Titan Biotech Ltd., New Delhi, India) (pH 5.8) supplemented with 3% (*w*/*v*) sucrose and 0.8% agar. The medium was autoclaved at 121 °C for 20 min, cooled to approximately 50 °C, and then supplemented under sterile conditions with filter-sterilized methyl jasmonate (MeJA) (ApoloScientific, Manchester, UK) stock solution. Cultures were maintained in a growth chamber at 24 ± 2 °C under a 16 h light/8 h dark photoperiod with a light intensity of 50 µmol m^−2^ s^−1^ [13].

After 30 days of cultivation, the aerial parts of in vitro-grown plants were harvested. After harvest, the aerial parts were gently rinsed with distilled water, blotted dry with paper towels and air-dried at room temperature, protected from direct sunlight, until constant weight. The dried material was then ground to a fine powder and stored in airtight containers at room temperature (in the dark) until extraction.

Wild plants were collected from natural populations located outside the city in mid-summer 2025. The wild plant material was identified as *Melilotus officinalis* (L.) based on observable morphological characteristics and comparison with a digitized reference specimen in the electronic herbarium of West Kazakhstan University [14]. At the time of collection, a formal voucher-deposition procedure was not included in the sampling protocol; therefore, no physical specimen from the original collection remains available for herbarium deposition. Photographs of the pressed plant material are provided as Supplementary Figure S1. The digitized record was used solely as a comparative taxonomic reference and does not constitute a voucher specimen from the present study.

### 2.2. Organic Acids

The organic acids profile was analyzed using ultra-fast liquid chromatography coupled with a photodiode array detector (UFLC-PDA; Shimadzu Corporation, Kyoto, Japan). Samples (2 g) were extracted by stirring with 25 mL of meta-phosphoric acid at 25 °C and 150 rpm for 25 min, followed by filtration through Whatman No. 4 paper [15]. Prior to analysis, the extract was further filtered through 0.20 µm nylon filters. The compounds were separated on a SphereClone C18 column (Phenomenex, Torrance, CA, USA) (5 μm, 250 × 4.6 mm i.d.), maintained at 35 °C. A 3.6 mM sulfuric acid solution was used as the eluent at a flow rate of 0.8 mL/min. Quantification was performed by relating the chromatographic peak areas to calibration curves constructed using commercial standards. The results were expressed in grams per 100 g of dry weight (g/100 g DW).

### 2.3. Phenolic Compounds Extraction

Hydroethanolic extracts (80:20, *v*/*v*) were prepared using a standardized laboratory method optimized for the extraction of both polar and non-polar compounds. Briefly, 2 g of dried sample were extracted with 80% ethanol under magnetic stirring for 1 h at room temperature. The mixture was then filtered through Whatman No. 4 filter paper. The extraction was repeated once (two extraction cycles total), and the filtrates were combined. The combined extract was concentrated under reduced pressure using a rotary evaporator (Büchi R-210, Flawil, Switzerland) at 40 °C and 100 mbar, and subsequently freeze-dried (FreeZone4.5, Labconco, Kansas City, MO, USA) [16,17].

### 2.4. Phenolic Compound Profiling by LC-MS/MS

The hydroethanolic extracts were re-dissolved in ethanol/water (20:80, *v*/*v*) to 10 mg/mL and filtered through 0.22 μm membranes. Phenolic compounds were analyzed by UHPLC (Vanquish, Thermo Scientific, Hemel Hempstead, UK) with diode-array and tandem mass spectrometric detection (HPLC-DAD-ESI-MS/MS), following the chromatographic conditions reported by Bessada et al. (2016) [16]. The MS system was an Orbitrap Exploris 120 mass spectrometer (Thermo Scientific, Hemel Hempstead, UK) with an electrospray ionization source operating in negative ionization mode with a voltage of 2.5 kV. Sheath gas, 50 a.u.; auxiliary gas, 10 a.u.; sweep gas, 1 a.u.; capillary temperature, 325 °C; and vaporizer temperature, 350 °C. Higher-energy collisional dissociation was performed at a normalized collision energy of 30% over a mass range of *m*/*z* 100–1500. Data acquisition and instrument control were performed using Xcalibur 4.6 software (Thermo Fisher Scientific). Compound identification was based on retention time, UV-Vis spectra and mass spectra compared with authentic standards; when standards were unavailable, tentative annotation relied on fragmentation profiles, evaluation of diagnostic neutral losses, and reference data from the literature [18].

Quantification was performed using external calibration curves and results were expressed as mg per g of extract. Calibration curves (1–80 mg/L) were established for: *p*-hydroxybenzoic acid (y = 19485x + 4004.4; R^2^ = 0.9999), *p*-coumaric acid (y = 31903x + 26136; R^2^ = 0.9982), ferulic acid (y = 39692x + 7655.8; R^2^ = 0.9999), kaempferol-3-*O*-rutinoside (y = 10230x + 3975.8; R^2^ = 0.9997), apigenin-7-*O*-glucoside, and quercetin-3-*O*-glucoside (y = 14381x + 1314.9; R^2^ = 0.9987), and kaempferol-3-*O*-glucoside (y = 15149x + 5241.5: R^2^ = 0.9998).

### 2.5. Antioxidant Activity

The antioxidant capacity of the hydroethanolic extracts was evaluated in vitro using two complementary assays: the thiobarbituric acid reactive substances (TBARS) assay and the ABTS radical cation decolorization assay. All analyses were performed in three independent experiments. Each assay was performed in three independent analytical runs, with three technical replicates per concentration. Freeze-dried hydroethanolic extracts were first dissolved in water to prepare a stock solution of 10 mg/mL, from which serial dilutions were prepared.

For the TBARS assay, lipid peroxidation was induced in pig brain (*Sus scrofa*) homogenates, and the formation of the malondialdehyde–thiobarbituric acid (MDA–TBA) complex was quantified as previously described [19], with absorbance measured at 532 nm. The percentage of inhibition was calculated using the equation:

(1) Inhibition (%) = (A − B)/A × 100

where A is the absorbance of the control and B is the absorbance of the sample. Results were expressed as EC_50_ values (µg/mL), defined as the extract concentration required to inhibit 50% of lipid peroxidation. Trolox (Sigma-Aldrich, St. Louis, MO, USA) was used as the reference antioxidant [16].

For the ABTS radical cation assay, the ABTS^+^-radical cation was generated by mixing equal volumes (1:1, *v*/*v*) of ABTS solution (7 mM) and potassium persulfate solution (4.95 mM) and incubating the mixture in the dark at room temperature for 16 h. The resulting ABTS^+^-solution was diluted with methanol until the absorbance at 734 nm reached 0.55. For the assay, 0.1 mL of each ethanolic plant extract was mixed with 3.9 mL of the diluted ABTS^+^-solution. The decrease in absorbance was recorded at 734 nm using a UV-1900i spectrophotometer (Shimadzu, Kyoto, Japan), with the ABTS^•+^ working solution without extract served as the control. Antioxidant activity was expressed as micromoles of Trolox equivalents per gram of extract (µmol TE/g extract), calculated from the Trolox calibration curve [18].

Trolox standard solutions were prepared at final concentrations of 0, 20, 40, and 80 µM and analysed under the same conditions as the hydroethanolic extracts. The calibration curve, obtained by plotting absorbance at 734 nm against Trolox concentration, was described by the equation A_734_ = −0.0043C + 0.5648, with R^2^ = 0.9862. The antioxidant capacity of the extracts was determined by interpolation from this calibration curve and expressed as μmol TE/g.

### 2.6. Antimicrobial Activity

Antimicrobial activity was evaluated using the broth microdilution method according to Clinical and Laboratory Standards Institute (CLSI) guidelines, with minor modifications [19,20]. Extracts were serially diluted twofold (10–0.15 mg/mL) in appropriate broth media, using 5% (*v*/*v*) DMSO as a solubilizing agent, a concentration widely recognized as non-inhibitory to microbial growth. The antimicrobial activity was evaluated against eight reference bacterial strains relevant to food safety, including five Gram-negative bacteria (*Enterobacter cloacae*, *Escherichia coli*, *Pseudomonas aeruginosa*, *Salmonella enterica*, and *Yersinia enterocolitica*) and three Gram-positive bacteria (*Bacillus cereus*, *Listeria monocytogenes*, and *Staphylococcus aureus*). Inocula were standardized to a 0.5 McFarland turbidity standard (~5 × 10^5^ CFU/mL) and the microplates were incubated at 37 °C for 24 h. Results were expressed as minimum inhibitory concentrations (MIC) and minimum bactericidal concentrations (MBC).

*Aspergillus brasiliensis* and *Aspergillus fumigatus* were tested under similar conditions, with results expressed as MIC and minimum fungicidal concentrations (MFC). Positive controls included ampicillin, streptomicin, and methicillin for antibacterial assays, and ketoconazole for antifungal assays.

The antimicrobial activity of the extracts was evaluated against eight reference bacterial strains, including five Gram-negative bacteria: *Enterobacter cloacae* ATCC 49741, *Escherichia coli* ATCC 25922, *Pseudomonas aeruginosa* ATCC 9027, *Salmonella enterica subsp. enterica serovar Enteritidis* ATCC 13076, and *Yersinia enterocolitica* ATCC 9610; and three Gram-positive bacteria: *Bacillus cereus* ATCC 11778, *Listeria monocytogenes* ATCC 19111, and *Staphylococcus aureus* ATCC 25923. The reference strains were purchased from Frilabo (Porto, Portugal).

### 2.7. Statistical Analysis

All analyses were performed in triplicate as technical replicates using a single bulked sample set for each study group (wild and in vitro-grown plants). Results, except for antimicrobial activity, are presented as mean ± standard deviation (SD). The SD reflects analytical/technical variability among replicate measurements and does not represent biological variation among individual plants. Consequently, no inferential statistical comparisons between wild and in vitro-grown plants were performed, and the observed differences are presented descriptively. All data processing and graphical visualization were performed using R software (version 4.5.3; R Foundation for Statistical Computing, Vienna, Austria). Because independent biological replicates were not included, the findings should be interpreted as preliminary and require validation using adequately replicated biological samples.

## 3. Results

### 3.1. Organic Acids

The organic acid profile of wild and in vitro-grown *Melilotus officinalis* is presented in Table 1. The total organic acid content was similar between wild (10.4 ± 1.4 g/100 g dw) and in vitro (10.5 ± 1.5 g/100 g dw) samples. However, notable differences were observed in the individual composition. Succinic acid was the predominant organic acid under both conditions (6.5 ± 0.9 g/100 g in wild vs. 4.8 ± 1.5 g/100 g in vitro). Oxalic acid was approximately threefold higher in the in vitro-grown material (5.78 ± 0.03 g/100 g DW) than in the wild-collected material (1.80 ± 0.10 g/100 g DW). Malic acid (1.8 ± 0.3 g/100 g) and citric acid (0.29 ± 0.06 g/100 g) were detected exclusively in wild samples.

### 3.2. Phenolic Compounds

Thirteen phenolic compounds were tentatively identified in hydroethanolic extracts of wild-collected and in vitro-grown *Melilotus officinalis* by LC–DAD–ESI–MS/MS analysis (Table 2).

Six phenolic acids were detected. Dihydroxybenzoic acid glucoside and its isomer (peaks 1 and 2, [M−H]^−^ at *m*/*z* 315), p-hydroxybenzoic acid hexoside (peak 3, [M−H]^−^ at *m*/*z* 299), and p-coumaric acid glucoside isomer (peak 8, [M−H]^−^ at *m*/*z* 325) were detected exclusively in wild samples (0.14 ± 0.01, 0.33 ± 0.03, 0.09 ± 0.01, and 2.37 ± 0.06 mg/g extract, respectively) [15]. p-Coumaric acid glucoside (peak 4) was present in both samples (1.36 ± 0.02 mg/g wild vs. 1.75 ± 0.01 mg/g in vitro). Ferulic acid hexoside (peak 5) was detected only in trace amounts in wild samples. Total phenolic acids were higher in wild samples (4.29 ± 0.07 mg/g) compared with the in vitro-grown material (1.75 ± 0.01 mg/g).

Three flavones were identified. Vicenin-3 (peak 9, [M−H]^−^ at *m*/*z* 563) was detected in both samples but was markedly higher in the in vitro-grown material (0.18 ± 0.01 mg/g wild vs. 2.07 ± 0.04 mg/g in vitro). Apigenin 6,8-di-C-pentoside isomers I and II (peaks 13 and 15, [M−H]^−^ at *m*/*z* 533) were detected exclusively in in vitro samples (1.50 ± 0.01 and 5.73 ± 0.08 mg/g, respectively).

Five flavonols were identified, all kaempferol and quercetin derivatives. Kaempferol-O-dirhamnoside-hexoside (peak 6), kaempferol-O-rhamnoside-rutinoside isomer I (peak 7), p-coumaric acid glucoside isomer (peak 8), quercetin-O-dirhamnoside-hexoside (peak 10), kaempferol-O-deoxyhexoside-hexoside (peak 12), and isorhamnetin-O-rhamnosyl-rutinoside (peak 14) were detected exclusively in wild samples. Kaempferol-O-rhamnoside-rutinoside (peak 11) was present in both samples but lower in the in vitro-grown material (2.37 ± 0.26 mg/g wild vs. 0.28 ± 0.02 mg/g in vitro; *p* < 0.05).

Total flavonoid content was markedly higher in in vitro samples (9.58 ± 0.06 mg/g) compared to wild (4.42 ± 0.24 mg/g), resulting in higher total phenolic content in the in vitro-grown material (11.33 ± 0.06 mg/g vs. 8.7 ± 0.3 mg/g in wild).

### 3.3. Antioxidant Activity

The antioxidant properties of hydroethanolic extracts obtained from wild-collected and in vitro-grown Melilotus officinalis are summarized in Table 3. Both extracts showed antioxidant activity, although their performance differed between the assays. The wild-collected extract showed an EC_50_ of 0.64 ± 0.01 mg/mL in the TBARS assay, whereas the EC_50_ of the in vitro-grown extract exceeded the highest concentration tested. In the ABTS assay, the wild-collected and in vitro-grown extracts showed values of 96.01 ± 0.05 and 85.64 ± 0.04 µmol TE/g extract, respectively. These results should be interpreted descriptively because the reported variability represents technical replicates rather than independent biological samples.

### 3.4. Antimicrobial Activity

The antimicrobial activity of wild and in vitro *Melilotus officinalis* hydroethanolic extracts against food-relevant bacterial and fungal pathogens is presented in Table 4. Both extracts demonstrated bacteriostatic and fungistatic properties with strain-specific patterns.

Among Gram-negative bacteria, the in vitro extract showed higher potency against *Yersinia enterocolitica* (MIC 1.25 mg/mL vs. 5 mg/mL for in vitro), while the wild extract was more effective against *Escherichia coli* (MIC 5 mg/mL in vitro vs. 10 mg/mL wild). Neither extract inhibited *Pseudomonas aeruginosa* at the highest tested concentration (MIC > 10 mg/mL). Against *Salmonella enterica* and *Enterobacter cloacae*, both extracts showed comparable activity (MIC 10 mg/mL).

Among Gram-positive bacteria, the wild extract was significantly more potent against *Listeria monocytogenes* (MIC 1.25 mg/mL in vitro vs. 10 mg/mL wild). Opposite, the in vitro extract was significantly more potent against *Bacillus cereus* (MIC 10 mg/mL in vitro vs. 2.5 mg/mL wild). Against *Staphylococcus aureus*, both extracts showed similar inhibitory concentrations.

For fungi, the in vitro extract exhibited higher potency against *Aspergillus brasiliensis* (MIC 1.25 mg/mL vs. 5 mg/mL for wild), while both extracts showed equal and limited activity against *Aspergillus fumigatus* (MIC 10 mg/mL).

## 4. Discussion

### 4.1. Organic Acids

The stability of total organic acid content between wild and in vitro samples, despite significant shifts in individual composition, reflects a cultivation-dependent metabolic reconfiguration. The predominance of succinic acid under both conditions is consistent with its central role in the TCA cycle in *Melilotus officinalis* [3]; however, its reduction in vitro may reflect altered carbon flux when plants are removed from natural soil environments and exposed to controlled laboratory conditions without the typical abiotic and biotic stressors of the wild [21]. The striking three-fold increase in oxalic acid in vitro may reflect a metabolic response to the specific nutrient composition of the Murashige & Skoog medium, which differs significantly from the semi-arid soils where wild specimens typically thrive [3,22]. The absence of malic and citric acids in vitro suggests that the observed absence may reflect lower accumulation under the tested in vitro conditions of these TCA intermediates, possibly due to the lack of soil microbial activity and natural nutrient cycling [23,24].

### 4.2. Phenolic Compounds

The results reveal a significant cultivation-dependent metabolic redirection in the phenylpropanoid pathway of *Melilotus officinalis.* The wild sample displayed greater diversity of phenolic acids, consistent with the literature reporting *p*-hydroxybenzoic and *p*-hydroxycinnamic acid derivatives as characteristic constituents of the species with key roles in plant defense [8]. In contrast, the in vitro profile was dominated by apigenin C-glycosides, with apigenin 6,8-di-C-pentoside isomer II alone (5.73 mg/g) exceeding the total flavonoid content of wild plants (4.42 mg/g).

The observed pattern may be consistent with MeJA-associated modulation of key phenylpropanoid pathway enzymes reported in other plant systems. MeJA acts as a signalling molecule that can influence the activity and expression of enzymes such as phenylalanine ammonia-lyase (PAL), chalcone synthase (CHS) and flavone synthase (FNS), thereby potentially directing biosynthetic flux toward flavone backbones such as apigenin at the expense of flavonol scaffolds (kaempferol/quercetin) in species where these responses have been directly demonstrated. In our study, we did not assess gene expression or enzyme activity, so this interpretation should be viewed as a plausible biochemical explanation rather than a confirmed mechanism. [8,25,26,27,28]. The sharp reduction of kaempferol-O-rhamnoside-rutinoside in vitro (from 2.37 to 0.28 mg/g) may be consistent with an effect of environmental conditions on flavonol accumulation [24]. The consistent presence of *p*-coumaric acid glucoside in both samples aligns with its role as a stable chemical marker for the species [29]. To the best of our knowledge, this is the first report on full individual phenolic compound characterization comparing wild and in vitro *Melilotus officinalis.*

The increased accumulation of apigenin di-C-pentoside isomers and vicenin-3 in MeJA-treated in vitro-grown plants may reflect elicitor-mediated modulation of the phenylpropanoid and flavone biosynthetic pathways. Methyl jasmonate is a well-known stress-related signaling molecule that can reallocate metabolic flux towards specialized metabolites. At the entry point to flavonoid biosynthesis, phenylalanine ammonia-lyase (PAL), cinnamate 4-hydroxylase (C4H), and 4-coumarate: CoA ligase (4CL) provide *p*-coumaroyl-CoA, whereas chalcone synthase (CHS) catalyzes the condensation of *p*-coumaroyl-CoA with malonyl-CoA to produce naringenin chalcone. Following isomerization by chalcone isomerase (CHI), flavone synthase (FNS) converts the flavanone intermediate naringenin into apigenin, which can subsequently undergo C-glycosylation to yield apigenin C-glycosides, including vicenin-type compounds. Therefore, the observed enrichment of apigenin di-C-pentosides and vicenin-3 is consistent with a possible MeJA-induced enhancement of flux through the CHS–CHI–FNS branch, together with the activity of C-glycosyltransferases responsible for the formation of stable C-glycosidic flavones. However, because gene expression and enzyme activities were not determined in the present study, this mechanism should be considered a biologically plausible interpretation rather than direct evidence. Future transcriptomic, RT-qPCR, and enzyme-activity analyses of PAL, 4CL, CHS, CHI, FNS, and relevant UDP-glycosyltransferase genes would help verify this proposed mechanism.

### 4.3. Antioxidant Activity

The contrasting antioxidant profiles between wild and in vitro extracts across the two assays reflect fundamental differences in their phenolic composition and the distinct mechanisms each assay evaluates. The superior performance of wild extracts in the TBARS assay is attributed to their richer content of kaempferol and quercetin glycosides, which are known to be more effective at inhibiting lipid peroxidation than apigenin C-glycosides. Structurally, kaempferol and quercetin possess a 3-hydroxyl group on the C-ring critical for hydrogen donation and radical stabilization, O-glycosidic linkages enabling membrane localization, and a catechol group on the B-ring facilitating metal chelation—all of which are absent in apigenin C-glycosides [19,30]. The EC_50_ of 0.64 mg/mL for the wild extract is consistent with reported values for potent antioxidant plant extracts used in functional food formulations [9].

The maintenance of high ABTS activity in in vitro extracts (85.64 μmol TE/g) despite their dramatically reduced TBARS performance suggests that apigenin 6,8-di-C-pentoside isomers are effective free radical scavengers in solution-based assays, even though their hydrophilic C-glycosidic linkage limits membrane localization and metal chelation. These observations suggest that antioxidant performance may depend on qualitative phenolic composition and assay mechanism rather than total phenolic content, and highlights the importance of using complementary assays for a comprehensive evaluation [5].

### 4.4. Antimicrobial Activity

The strain-specific antimicrobial patterns observed reflect differences in phenolic composition between the two extracts and the distinct susceptibility mechanisms of each pathogen. The superior activity of wild extracts against *L. monocytogenes* and *Y. enterocolitica* may be associated with their higher content of kaempferol and quercetin glycosides, which are known to disrupt bacterial cell membrane integrity and inhibit DNA gyrase activity in Gram-positive and certain Gram-negative species [8]. Conversely, the higher potency of in vitro extracts against *A. brasiliensis* may be linked to the elevated concentrations of apigenin 6,8-di-C-pentoside isomers and vicenin-3, which have been reported to disrupt fungal cell membranes and inhibit specific metabolic pathways [31].

The resistance of *P. aeruginosa* to both extracts is consistent with the literature, as this pathogen possesses structurally complex biofilms and active efflux pumps that effectively remove phenolic compounds [5]. Similarly, the limited activity against *A. fumigatus* (MIC 10 mg/mL for both extracts) suggests inherent resistance mechanisms related to cell wall structural complexity. Overall, the complementary antimicrobial profiles of wild and in vitro extracts suggest that the choice of material should be guided by the target pathogen and the intended application, whether in food preservation, nutraceutical formulations or other uses of plant-derived bioactive extracts.

### 4.5. Safety and Toxicological Considerations

The application of *Melilotus officinalis* extracts as bioactive ingredients in foods, supplements or phytomedicines requires careful consideration of two key safety aspects: the presence of coumarin and the cultivation-dependent accumulation of oxalic acid.

Coumarin is a characteristic secondary metabolite of *Melilotus officinalis*, with reported concentrations in dried aerial parts ranging from 0.3 to 0.9% (3–9 mg/g dw), with the European Pharmacopoeia establishing a minimum content of 0.3% as a quality standard for the herbal drug [32]. The European Food Safety Authority (EFSA) established a Tolerable Daily Intake (TDI) of 0.1 mg coumarin/kg body weight per day, based on hepatotoxicity data from long-term animal studies; this value was subsequently confirmed following a comprehensive review of human metabolism data [13,33]. For a 60 kg adult, this corresponds to a maximum safe intake of 6 mg coumarin per day. Hepatotoxic effects in humans have been associated primarily with chronic intake exceeding 25–90 mg/day, though individual susceptibility varies depending on CYP2A6 polymorphism status [33]. At the expected use levels as a food ingredient or natural preservative, *Melilotus officinalis* extracts are unlikely to exceed the TDI; however, standardized monitoring of coumarin content is recommended, particularly for concentrated extracts and food supplement applications [12].

Beyond coumarin, the results of the present study revealed a striking three-fold increase in oxalic acid content in in vitro-grown samples (5.78 ± 0.03 g/100 g dw) compared to wild plants (1.80 ± 0.10 g/100 g dw), which represents an original toxicological finding of direct practical relevance. Dietary oxalates are known antinutrients that bind calcium and other divalent minerals, reducing their bioavailability, and promoting calcium oxalate crystal formation in the urinary tract upon chronic high intake [15]. Although oxalic acid is a normal end product of plant metabolism, elevated concentrations—as observed in the in vitro material—may pose a risk of nephrolithiasis under conditions of regular high-dose consumption [15,34] Notably, conventional food processing methods such as boiling or soaking can significantly reduce soluble oxalate content through leaching, which may partially mitigate this concern for processed plant derived products [35].

The present data do not permit a safety assessment of the extracts because coumarin content, extract exposure, bioavailability, and toxicological endpoints were not evaluated. The observed oxalic-acid concentrations and the known occurrence of coumarin in *Melilotus officinalis* indicate that targeted safety assessment, including coumarin quantification, is required before food or supplement applications can be considered.

### 4.6. Limitations of the Study

This study has several limitations that should be considered when interpreting the findings. First, a conventional herbarium voucher specimen from the original wild plant collection was not retained and therefore could not be deposited retrospectively. Photographs of the pressed plant material and the available collection metadata are provided as supplementary documentation; however, these materials do not replace a deposited herbarium voucher. Second, wild plants were collected from a single location and season in northern Kazakhstan, and the results may not fully represent the broader ecological variation of *Melilotus officinalis* across its native range. Third, although all analyses were performed in triplicate, the replicates represent technical repeats of one bulked sample per cultivation system rather than independent biological replicates, which constrains the strength of statistical inference. Fourth, only one in vitro protocol including MeJA supplementation was evaluated; future work should compare non-elicited cultures and alternative elicitation regimes to disentangle the specific contribution of MeJA from the general effects of tissue culture conditions.

## 5. Conclusions

This study shows that in vitro cultivation of *Melilotus officinalis* substantially modifies its metabolic profile and bioactivity, yielding a viable but more specialized source of natural bioactive ingredients. In vitro cultures displayed higher total phenolic and flavonoid content, mainly due to apigenin 6,8-di-C-pentoside isomers and vicenin-3, while wild plants retained greater diversity of phenolic acids and kaempferol/quercetin glycosides. These compositional differences were reflected in biological activity: wild extracts were more effective in inhibiting lipid peroxidation, whereas both sources showed comparable radical scavenging capacity and complementary, strain-dependent antimicrobial profiles. Compositional data also highlight the need to monitor coumarin content and to account for the elevated oxalic acid levels in in vitro material when considering downstream applications, particularly for concentrated extracts. Overall, wild and in vitro-grown *Melilotus officinalis* represent complementary sources of bioactive compounds, with the choice of cultivation method offering a means to tailor phytochemical profiles to specific biochemical and safety-related requirements.

## Figures and Tables

**Table 1:** Organic acids profile of the analyzed wild and in vitro *Melilotus officinalis* samples.

Samples	*Melilotus officinalis* (Wild)	*Melilotus officinalis* (In Vitro)
Organic acids (g/100g DW)		
Oxalic acid	1.80 ± 0.10	5.78 ± 0.03
Malic acid	1.80 ± 0.30	n.d.
Citric acid	0.29 ± 0.06	n.d.
Succinic acid	6.50 ± 0.90	4.80 ± 1.50
Total organic acids	10.4 ± 1.40	10.5 ± 1.50

n.d.—non detected. Values are mean ± SD (n = 3).

**Table 2:** Retention times, λ_max_ values, MS/MS data, and quantification (mean ± SD) of the phenolic compounds of wild and in vitro *Melilotus officinalis* hydroethanolic extracts.

Peak	Rt (min)	λmax (nm)	[M−H]^−^	MS^2^	Assignment	mg/g Extract	
						*Melilotus officinalis* Wild	*Melilotus officinalis* (In Vitro)
1	4.56	324	315	153, 152, 109, 108	Dihydroxybenzoic acid glucoside ^1^	0.14 ± 0.01	nd
2	5.86	280	315	153, 109	Dihydroxybenzoic acid glucoside isomer ^1^	0.33 ± 0.03	nd
3	6.53	292	299	137, 93	*p*-hydroxybenzoic acid hexoside ^1^	0.09 ± 0.01	nd
4	7.23	nd	325	163, 119	*p*-coumaric acid glucoside^2^	1.36 ± 0.02	1.75 ± 0.01
5	8.73	nd	355	193	Ferulic acid hexoside ^3^	tr	nd
6	10.76	264, 348	885	739, 431, 285	Kaempferol-*O*-dirhamnoside-hexoside ^4^	0.98 ± 0.01	nd
7	11.78	264, 348	739	593, 431, 285, 283	Kaempferol-*O*-rhamnoside-rutinoside	0.12 ± 0.01	nd
8	12.86	nd	325	163, 119	*p*-coumaric acid glucoside isomer ^2^	2.37 ± 0.06	nd
9	13.51	nd	563	473, 443, 383, 353	Vicenin-3 ^5^	0.18 ± 0.01	2.07 ± 0.04
10	13.68	256, 356	755	609, 447, 301	Quercetin-*O*-dirhamnoside-hexoside ^6^	0.54 ± 0.01	nd
11	15.35	264, 348	739	593, 431, 285, 283	Kaempferol-*O*-rhamnoside-rutinoside ^4^	2.37 ± 0.26	0.28 ± 0.02
12	15.66	nd	593	447, 430, 285, 283	Kaempferol-*O*-deoxyhexoside-hexoside ^7^	0.07 ± 0.01	nd
13	16.06	272, 336	533	473, 443, 383, 353	Apigenin 6,8-di-*C*-pentoside ^5^	nd	1.50 ± 0.01
14	16.63	nd	769	623, 315	Isorhamnetin-*O*-rhamnosyl-rutinoside ^6^	0.16 ± 0.02	nd
15	16.68	272, 336	533	473, 443, 383, 353	Apigenin 6,8-di-*C*-pentoside isomer ^5^	nd	5.73 ± 0.08
					Total phenolic acids	4.29 ± 0.07	1.75 ± 0.01
					Total flavonoids	4.42 ± 0.24	9.58 ± 0.06
					Total phenolic compounds	8.7 ± 0.3	11.33 ± 0.06

nd—non detected; tr—traces; When standards were not available, compound annotation was performed based on comparison of fragmentation pattern with that of literature, and evaluation of diagnostic neutral losses. Standard curves used for quantification: *p*-hydroxybenzoic ^1^, coumaric ^2^, ferulic ^3^, kaempferol-3-*O*-rutinoside ^4^, apigenin-7-*O*-glucoside ^5^, and quercetin-3-*O*-glucoside ^6^, and kaempferol-3-*O*-glucoside ^7^.

**Table 3:** Antioxidant properties of wild and in vitro *Melilotus officinalis* hydroethanolic extracts.

	*Melilotus officinalis* (Wild)	*Melillotus officinalis* (In Vitro)
TBARS (EC_50_ = mg/mL)	0.64 ± 0.01	>10
ABTS (μmol TE/g)	96.01 ± 0.05	85.64 ± 0.04

Values are mean ± SD (*n* = 3). For TBARS, EC_50_ for the in vitro extract was higher than the maximum tested concentration (EC_50_ > 10 mg/mL), so no *t*-test was performed. Trolox EC_50_ values (positive control): 5.4 ± 0.3 µg/mL.

**Table 4:** Antimicrobial activity of the wild and in vitro *Melilotus officinalis* hydroethanolic extracts.

	Sample				Positive Control					
	*Melilotus officinalis* (Wild)		*Melilotus officinalis* (In Vitro)		Streptomycin 1 mg/mL		Methicillin 1 mg/mL		Ampicillin 10 mg/mL	
**Antibacterial Activity (MIC/MBC, mg/mL)**										
	**MIC**	**MBC**	**MIC**	**MBC**	**MIC**	**MBC**	**MIC**	**MBC**	**MIC**	**MBC**
Gram-negative bacteria										
*Enterobacter cloacae*	5	>10	10	>10	0.007	0.007	n.t.	n.t	0.15	0.15
*Escherichia coli*	10	>10	5	>10	0.01	0.01	n.t.	n.t.	0.15	0.15
*Pseudomonas aeruginosa*	>10	>10	>10	>10	0.06	0.06	n.t.	n.t.	0.63	0.63
*Salmonella enterica*	10	>10	10	>10	0.007	0.007	n.t.	n.t.	0.15	0.15
*Yersinia enterocolitica*	1.25	>10	5	>10	0.007	0.007	n.t.	n.t.	0.15	0.15
Gram-positive bacteria										
*Bacillus cereus*	2.5	>10	10	>10	0.007	0.007	n.t.	n.t.	n.t.	n.t.
*Listeria monocytogenes*	10	>10	1.25	>10	0.007	0.007	n.t.	n.t.	0.15	0.15
*Staphylococcus aureus*	2.5	>10	5	>10	0.007	0.007	0.007	0.007	0.15	0.15
Antifungal activity (MIC/MFC, mg/mL)										
	*Aspergillus brasiliensis*					*Aspergillus fumigatus*				
	**MIC**		**MFC**			**MIC**		**MFC**		
*Melilotus officinalis* (wild)	5		>10			10		>10		
*Melilotus officinalis* (in vitro)	1.25		>10			10		>10		
Ketoconazole	0.06		0.125			0.5		1		

MIC, Minimum Inhibitory Concentration; MBC, Minimum Bactericidal Concentration; MFC, Minimum Fungicidal Concentration; n.t.—not tested.

## Data Availability

The original contributions presented in this study are included in the article. Further inquiries can be directed to the corresponding author.

## Supplementary Materials

The following supporting information can be downloaded at: https://www.mdpi.com/article/10.3390/life16091488/s1, Figure S1: *Melilotus officinalis* (L.).

## References

[1] Chauhan K., Rao A. (2024). Clean-label alternatives for food preservation: An emerging trend. Heliyon.

[2] Shahidi F., Ambigaipalan P. (2015). Phenolics and polyphenolics in foods, beverages and spices: Antioxidant activity and health effects—A review. J. Funct. Foods.

[3] Nogues I., Passatore L., Bustamante M.Á., Pallozzi E., Luz J., Traquete F., Ferreira A.E.N., Sousa Silva M., Cordeiro C. (2023). Cultivation of *Melilotus officinalis* as a source of bioactive compounds in association with soil recovery practices. Front. Plant Sci..

[4] Pastorino G., Marchetti C., Borghesi B., Cornara L., Ribulla S., Burlando B. (2017). Biological activities of the legume crops *Melilotus officinalis* and *Lespedeza capitata* for skin care and pharmaceutical applications. Ind. Crops Prod..

[5] Babich O., Frolov A., Bakhtiyarova A.K., Motorzhina A.V., Alhajje K., Orlova A., Tsvetkova E.V., Levada K.V., Ivanova S., Sukhikh S. (2026). Study of cytotoxic, anti-inflammatory, antioxidant, and antibacterial properties of *Melilotus officinalis* extracts. Biocatal. Agric. Biotechnol..

[6] Liu Y.-T., Gong P.-H., Xiao F.-Q., Shao S., Zhao D.-Q., Yan M.-M., Yang X.-W. (2018). Chemical constituents and antioxidant, anti-inflammatory and anti-tumor activities of *Melilotus officinalis* (Linn.) Pall. Molecules.

[7] Toiu A., Vlase A.-M., Vlase L., Casian T., Pârvu A.E., Oniga I. (2025). In vivo investigation of cardioprotective effects of *Melilotus officinalis* and *Melilotus albus* aerial parts extracts for potential therapeutic application. Plants.

[8] Yildiz İ., Başar Y., Erenler R., Alma M.H., Calimli M.H. (2024). A phytochemical content analysis and antioxidant activity evaluation using a novel method on *Melilotus officinalis* flower. S. Afr. J. Bot..

[9] Toiu A., Vlase L., Gheldiu A., Vodnar D., Oniga I. (2017). Evaluation of the antioxidant and antibacterial potential of compounds from *Ajuga reptans* extracts. Farmacia.

[10] Wink M. (2003). Evolution of secondary metabolites from an ecological and molecular phylogenetic perspective. Phytochemistry.

[11] Fazili M.A., Bashir I., Ahmad M., Yaqoob U., Geelani S.N. (2022). In vitro strategies for the enhancement of secondary metabolite production in plants: A review. Bull. Natl. Res. Cent..

[12] Lončar M., Jakovljević M., Šubarić D., Pavlić M., Buzjak Služek V., Cindrić I., Molnar M. (2020). Coumarins in food and methods of their determination. Foods.

[13] Zhakupova G., Sagandyk A., Tultabayeva T., Muldasheva A., Makangali K., Akhmetzhanova A. (2026). Stress-associated phenylpropanoid metabolism and nutritional composition in wild vs. MeJA-elicited in vitro *Hypericum perforatum* and *Portulaca oleracea*. Metabolites.

[14] West Kazakhstan University Electronic Herbarium: *Melilotus officinalis* (L.) Lam. https://eherbarium.wku.edu.kz/ru/obshchaya-informatsiya/details/1/31.

[15] Barros L., Pereira C., Ferreira I.C.F.R. (2013). Optimized analysis of organic acids in edible mushrooms from Portugal by ultra fast liquid chromatography and photodiode array detection. Food Anal. Methods.

[16] Bessada S.M.F., Barreira J.C.M., Santos J., Costa C., Pimentel F.B., Bessa M.J., Teixeira J.P., Oliveira M.B.P. (2017). Evaluation of the cytotoxicity (HepG2) and chemical composition of polar extracts from the ruderal species *Coleostephus myconis* (L.) Rchb.f. J. Toxicol. Environ. Health Part A.

[17] Sagandyk A.T., Liberal Â., da Silveira T.F., Alves M.J., Ferreira I.C., Zhakupova G.N., Makangali K., Tultabayeva T.C., Barros L. (2024). Nutritional, phytochemical, and bioactive prospects of black chokeberry (*Aronia melanocarpa*) and saskatoon berry (*Amelanchier ovalis*) grown in the Republic of Kazakhstan. Appl. Food Res..

[18] Sweetlove L.J., Beard K.F., Nunes-Nesi A., Fernie A.R., Ratcliffe R.G. (2010). Not just a circle: Flux modes in the plant TCA cycle. Trends Plant Sci..

[19] Roriz C.L., Barros L., Carvalho A.M., Santos-Buelga C., Ferreira I.C.F.R. (2014). *Pterospartum tridentatum*, *Gomphrena globosa* and *Cymbopogon citratus*: A phytochemical study focused on antioxidant compounds. Food Res. Int..

[20] Pires T.C., Dias M.I., Barros L., Alves M.J., Oliveira M.B.P., Santos-Buelga C., Ferreira I.C. (2018). Antioxidant and antimicrobial properties of dried Portuguese apple variety (*Malus domestica* Borkh. cv Bravo de Esmolfe). Food Chem..

[21] Chaves N., Santiago A., Alías J.C. (2020). Quantification of the antioxidant activity of plant extracts: Analysis of sensitivity and hierarchization based on the method used. Antioxidants.

[22] Li P., Liu C., Luo Y., Shi H., Li Q., Chu P., Li X., Yang J., Fan W. (2022). Oxalate in plants: Metabolism, function, regulation, and application. J. Agric. Food Chem..

[23] Ditta A., Rout G.R., Das A.B. (2013). Salt tolerance in cereals: Molecular mechanisms and applications. Molecular Stress Physiology of Plants.

[24] Zhang T., Peng J.T., Klair A., Dickinson A.J. (2023). Non-canonical and developmental roles of the TCA cycle in plants. Curr. Opin. Plant Biol..

[25] Halder M., Sarkar S., Jha S. (2019). Elicitation: A biotechnological tool for enhanced production of secondary metabolites in hairy root cultures. Eng. Life Sci..

[26] Wang X.X., Li X.B., Peng C.S. (2019). Comprehensive mass spectrum analysis of two flavone-6,8-C-di-glycosides and its application by high resolution electrospray ionization tandem mass spectroscopy in both negative and positive ion modes. Zhongguo Zhongyao Zazhi (China J. Chin. Mater. Medica).

[27] Wasternack C., Song S. (2017). Jasmonates: Biosynthesis, metabolism, and signaling by proteins activating and repressing transcription. J. Exp. Bot..

[28] Li S., Xiao L., Chen M., Cao Q., Luo Z., Kang N., Jia M., Chen J., Xiang M. (2022). The involvement of the phenylpropanoid and jasmonate pathways in methyl jasmonate-induced soft rot resistance in kiwifruit (*Actinidia chinensis*). Front. Plant Sci..

[29] Bradic J., Petrovic A., Nikolic M., Nedeljkovic N., Andjic M., Kladar N., Bolevich S., Jakovljevic V., Kocovic A. (2024). Newly developed semi-solid formulations containing *Melilotus officinalis* extract: Characterization, assessment of stability, safety, and anti-inflammatory activity. Pharmaceutics.

[30] Jan R., Khan M., Asaf S., Lubna, Asif S., Kim K.-M. (2022). Bioactivity and therapeutic potential of kaempferol and quercetin: New insights for plant and human health. Plants.

[31] Li Z., Zhou J., Ji L., Liang Y., Xie S. (2023). Recent advances in the pharmacological actions of apigenin, its complexes, and its derivatives. Food Rev. Int..

[32] European Medicines Agency (EMA) (2016). Final Assessment Report on Melilotus Officinalis (L.) Lam., Herba.

[33] European Food Safety Authority (EFSA) (2008). Coumarin in flavourings and other food ingredients with flavouring properties—Scientific opinion of the Panel on Food Additives, Flavourings, Processing Aids and Materials in Contact with Food (AFC). EFSA J..

[34] Siener R. (2021). Nutrition and kidney stone disease. Nutrients.

[35] Huynh N.K., Nguyen H.V.H. (2022). Effects of processing on oxalate contents in plant foods. J. Food Compos. Anal..

